# Simulating “soft” electronics

**DOI:** 10.1186/1758-2946-6-S1-O19

**Published:** 2014-03-11

**Authors:** Tim Clark

**Affiliations:** 1 Computer-Chemie-Centrum, Department Chemie und Pharmazie, Friedrich-Alexander-Universität Erlangen-Nürnberg, Nägelsbachstraße 25, 91052 Erlangen, Germany

## 

Simulating electronic devices built from flexible organic molecules requires both adequate conformational sampling and a reliable quantum mechanical description of the electronics of the system.

The former is best achieved using classical (force field) molecular-dynamics simulations, from which individual geometries (“snapshots”) can be used for subsequent quantum mechanical calculations.

As the repeating unit in the classical simulations typically involves thousands of atoms, the quantum mechanical techniques must also be able to handle many thousands of atoms quickly and effectively on modern parallel hardware.

The newly developed EMPIRE program has been used in such a scheme to perform simulations on self-assembled-monolayer field-effect transistors (SAMFETs). Calculations of the size needed require novel interpretation and post-processing techniques based on local properties calculated on grids, rather than the more traditional population analyses.

The results of simulations using the techniques described above will be presented and the algorithms and parallelization strategies implemented to be able to calculate as many as 100,000 atoms on 1,024 cores.

